# Clinical Characteristics and Healthcare Resource Utilization for Patients with Mucopolysaccharidosis II (MPS II) in the United States: A Retrospective Chart Review

**DOI:** 10.36469/jheor.2022.33801

**Published:** 2022-05-12

**Authors:** Olulade Ayodele, Kersten Müller, Solmaz Setayeshgar, David Alexanderian, Karen S. Yee

**Affiliations:** 1 Takeda Development Center, Inc., Lexington, MA; 2 ICON plc, Vancouver, British Columbia, Canada; 3 Takeda Development Center, Inc., Cambridge, MA

**Keywords:** burden of illness, MPS II, Hunter syndrome, lysosomal storage disease, enzyme replacement therapy, healthcare resource utilization

## Abstract

**Background:** Mucopolysaccharidosis II (MPS II; Hunter syndrome) is a rare, X-linked, life-limiting lysosomal storage disease characterized by a deficiency in the activity of the enzyme iduronate-2-sulfatase. Accumulation of glycosaminoglycans in tissues and organs throughout the body causes cellular damage, leading to multisystemic disease manifestations. Patients generally require multidisciplinary care across a wide range of specialties. **Objectives:** The aims of this study were to assess the healthcare needs of patients with MPS II and to explore the impact of treatment on disease burden and healthcare resource utilization. **Methods:** A retrospective review of medical charts from 19 US sites was performed. Data were analyzed from 140 male patients diagnosed with MPS II (defined as a documented deficiency in iduronate-2-sulfatase) between 1997 and 2017. The prevalence and age at onset of clinical manifestations and extent and frequency of healthcare resource use were evaluated. **Results:** Of the patients in this study, 77.1% had received enzyme replacement therapy with intravenous idursulfase and 62.1% had cognitive impairment. The clinical burden among patients was substantial: almost all patients had ear, nose, and throat abnormalities (95.7%); musculoskeletal abnormalities (95.0%); and joint stiffness or abnormalities (90.7%). Of the most prevalent disease manifestations, facial dysmorphism and hepatosplenomegaly were documented the earliest (median age at first documentation of 3.8 years in both cases). Hospitalizations, emergency department visits, and outpatient visits were reported for 51.2%, 58.5%, and 93.5% of patients, respectively, with a frequency of 0.1, 0.2, and 3.0 per patient per year, respectively. Surgery was also common, with 91.1% of patients having undergone at least 1 surgical procedure. The clinical burden and prevalence and frequency of resource use were generally similar in patients who had received enzyme replacement therapy and in those who had not. **Conclusions:** These results add to our understanding of the natural history of MPS II and indicate that the disease burden and healthcare needs of patients with this progressive disease are extensive. Increased understanding of disease burden and resource use may enable the development of models of healthcare resource utilization in patients with MPS II and contribute to improvements in disease management and patient care.

## BACKGROUND

Mucopolysaccharidosis II (MPS II; also known as Hunter syndrome; OMIM 309900) is a rare, X-linked, life-limiting lysosomal storage disease characterized by a deficiency in the activity of the enzyme iduronate-2-sulfatase (I2S). The estimated incidence of the disease is 0.10-2.16 per 100 000 live births,^1^ and, although MPS II predominantly affects males, a small number of affected females have been reported.^2–5^

Deficient activity of I2S is associated with accumulation of the glycosaminoglycans (GAGs) heparan sulfate and dermatan sulfate in tissues and organs throughout the body.^6,7^ Cellular damage occurs as these materials accumulate within the cell and, over time, may result in organ dysfunction and multiple organ failure.^7^ Consequently, MPS II is a multisystemic and progressive disease, and its somatic signs and symptoms typically emerge in the first few years of life.^6^ Clinical manifestations vary in both presentation and severity and may include coarse facial features, obstructive and restrictive respiratory disease, ear, nose, and throat (ENT) abnormalities, musculoskeletal abnormalities, and organ dysfunction.^6–9^ Approximately two-thirds of patients have the neuronopathic form of the disease, which is defined by progressive central nervous system involvement and cognitive impairment.^6,9^ Patients with central nervous system involvement typically experience more rapid disease progression and usually survive until the second decade of life; however, patients with the non-neuronopathic form of the disease may survive into their fifth or sixth decade.^6,10,11^ All patients, regardless of cognitive involvement, experience significant somatic manifestations, high disease burden, and reduced quality of life.^8,9,12^

As a result of the multisystemic nature of MPS II, effective management of this disease requires concerted multidisciplinary care across a wide range of specialties, including cardiology (eg, echocardiograms), otorhinolaryngology (eg, tonsillectomy/adenoidectomy and otologic/audiologic tests), orthopedic procedures (eg, correction of spinal deformities and imaging), and physical therapy (eg, assistance with musculoskeletal abnormalities, joint strength, and stiffness).^13,14^ A few studies in Europe^15,16^ and the United States^17^ have documented the healthcare resource utilization (HRU) associated with this chronic and debilitating disease. A recent study demonstrated that HRU among patients with MPS II in the United States is extensive; 40% of patients had received emergency care, 44% had undergone day surgeries, and 58% had been hospitalized within a 1-year period.^17^

Disease-specific treatment is available in the form of a weekly intravenous infusion of enzyme replacement therapy (ERT) with a recombinant form of human I2S (idursulfase [marketed as ELAPRASE®], Takeda Pharmaceuticals U.S.A. Inc., Lexington, Massachusetts). Idursulfase was approved for patients with MPS II in the United States in 2006 and in Europe and Japan the following year. It is the current standard of care and has been demonstrated to improve or to stabilize many of the somatic symptoms associated with MPS II.^18^ Idursulfase does not, however, cross the blood-brain barrier in therapeutic quantities, so it is not expected to address neurological aspects of the disease.^18,19^ Hematopoietic stem cell transplants (HSCTs), including bone marrow grafts, have also been used as a therapeutic approach in some patients with MPS II; however, use has been limited by high risks of transplantation-related morbidity and mortality.^20,21^

Although our understanding of the multidisciplinary care required by patients with MPS II has improved, there remains a need to gain a greater understanding of the healthcare needs in patients and, in particular, to assess the impact of intravenous ERT. Therefore, the aim of this real-world study was to understand the clinical burden and HRU associated with the treatment of patients with MPS II in the United States over a 20-year period (1997-2017). We report the clinical characteristics and HRU of the patient population in this study and explore the impact of treatment status on disease burden and resource use.

## METHODS

### Study Design and Population

A retrospective medical chart review of patients with MPS II was conducted at 19 sites in the United States to quantify the disease burden and associated HRU in this population. Study sites were specialist clinics, and study investigators were specialists in the treatment of patients with MPS II at major US medical sites. Sites were selected based on responses to a feasibility assessment questionnaire that assessed the interests of the study investigator, the number of eligible patients with MPS II, the ethics approval process at the site, and the capacity of the site staff to perform data extraction. Eligible individuals and their medical charts were identified by participating physicians and site staff. Male patients of any age at participating sites with a diagnosis of MPS II, defined as a documented deficiency of I2S between 1997 and 2017, were eligible for inclusion in the overall analysis population (n=140). Evidence of I2S deficiency included a lab report and/ or genetic analysis, phone report or other communication (more than one type of evidence could be documented per patient). Due to the rarity of the disease, no other inclusion or exclusion criteria were implemented; patients were included irrespective of ERT status, cognitive involvement, or previous HSCT. Data were derived from living and deceased patients.

For HRU analyses, 17 patients were excluded because they either had only 1 documented visit or their data had been collected at a site where more than 25% of resource utilization was characterized as unknown for at least 1 of the 3 core variables (surgery, hospitalization, and outpatient visits). Therefore, the analysis population for resource use analyses was 123 patients. For analyses of the annual frequency of resource use, 1 additional patient was excluded (analysis population, n=122).

### Ethics Approval and Consent to Participate

This study was approved by the Western Institutional Review Board (IRB) and by all relevant local IRBs. Waivers of consent for the data collection were granted by all IRBs.

### Data Collection and Analyses

An online database was developed to capture relevant information from the charts of all included patients. Data were collected from as early as available in patients’ charts until the last date of data entry, loss to follow-up, or death, whichever occurred first. Data were entered into an electronic case report form (DataTrak) and cleaned by the authors via automated queries, manual review and reclassification. If potential data entry errors in the electronic case report form were detected or clarifications were required, queries were posed to the relevant site through the online system. Sites then made corrections to the entered data as necessary. Where appropriate, free-text entries for clinical characteristics, outcomes or resource use that had been listed as “other” were reclassified into existing categories. New categories were created for responses that were mentioned frequently in free-text responses. All entries that were not recategorized remained as “other.”

All analyses were performed using the SAS software version 9.4 (SAS Institute Inc, Cary, North Carolina). Data for patient demographics, clinical characteristics, clinical outcomes, and resource utilization were summarized descriptively using means, standard deviations (SD), medians, and interquartile ranges for continuous variables, and using numbers and percentages for categorical variables. Data were analyzed in aggregate and also stratified by ERT status. Patients were categorized as “treated” if they had ever received ERT (at least 1 treatment with idursulfase documented in their chart). Patients were characterized as having cognitive impairment if cognitive delay was documented in their chart at any time during the study, with or without formal testing.

For HRU, both the proportion of patients with any use of a specific resource and the annual frequency of use were reported. To calculate the average frequency of resource use per patient, the total number of resource use occurrences (in all patients included in the analysis, n=122) was divided by the total number of follow-up years (defined as the period between the first and last documented visit). A sensitivity analysis was also performed to evaluate any impact of excluding patients who had received an HSCT on clinical and resource use findings.

## RESULTS

### Patient Characteristics and Treatment Status

The characteristics of the overall patient population (n=140) are summarized in Table 1. Based on their last known vital status, 122 patients were alive (mean age, 11.5 years) and 18 were deceased (mean age, 12.5 years) at the time of data entry. Patients were from specialist sites in 14 of 50 US states, providing good geographic coverage. MPS II was diagnosed at a mean age of 3.8 years (range, 1.2 months before birth to 59.7 years), and 53 patients (37.9%) had a family history of MPS II. Overall, 87 patients (62.1%) had a record of cognitive impairment documented in their charts.

**Table 1.** Summary of Patient Characteristics for All Patients and Stratified by ERT Status

	**All Patients (n=140)**	**Received ERT (n=108)**	**Never Received ERT (n=32)**
Alive, n (%)^a^	122 (87.1)	100 (92.6)	22 (68.8)
Last documented age, years (alive)			
Mean (SD)	11.5 (7.7)	11.3 (6.4)	12 (12.4)
Median (range)	9.8 (0.6-62.5)	9.9 (0.8-28.8)	8.0 (0.6-62.5)
Deceased, n (%)	18 (12.9)	8 (7.4)	10 (31.3)
Last documented age, years (deceased)			
Mean (SD)	12.5 (4.9)	15.1 (3.9)	10.4 (4.8)
Median (range)	13.4 (3.3-20.2)	15.3 (9.1-20.2)	12.2 (3.3-16.2)
Cause of death, n (%)			
Related to MPS II	7 (38.9)	3 (37.5)	4 (40.0)
Unclear if related to MPS II	5 (27.8)	2 (25.0)	3 (30.0)
Unknown	6 (33.3)	3 (37.5)	3 (30.0)
Age at diagnosis, years^b^	n=136	n=105	n=31
Mean (SD)	3.8 (5.8)	3.5 (3.4)	4.8 (10.4)
Median (range)^c,d^	2.8 (-0.1-59.7)	2.8 (-0.1-17.8)	2.8 (0.2-59.7)
Cognitive impairment, n (%)	87 (62.1)	69 (63.9)	18 (56.3)
Family history of MPS II, n (%)^e^	53 (37.9)	40 (37.0)	13 (40.6)
Sibling(s) with MPS II^f^	33 (62.3)	22 (55.0)	11 (84.6)
Uncle(s) with MPS II^f^	15 (28.3)	12 (30.0)	3 (23.1)
Ethnicity, n (%)			
Caucasian	76 (54.3)	54 (50.0)	22 (68.8)
Black/African American	25 (17.9)	18 (16.7)	7 (21.9)
Hispanic/Latino	21 (15.0)	20 (18.5)	1 (3.1)
Mixed	7 (5.0)	6 (5.6)	1 (3.1)
Asian	5 (3.6)	4 (3.7)	1 (3.1)
Other	5 (3.6)	5 (4.6)	0 (0.0)
Unknown	1 (0.7)	1 (0.9)	0 (0.0)
Insurance status			
Private	58 (41.4)	43 (39.8)	15 (46.9)
Medicaid	57 (40.7)	46 (42.6)	11 (34.4)
Multi-insurance	13 (9.3)	10 (9.3)	3 (9.4)
Other	9 (6.4)	7 (6.5)	2 (6.3)
Unknown	2 (1.4)	2 (1.9)	0 (0.0)
None	1 (0.7)	0 (0.0)	1 (3.1)

Abbreviations: ERT, enzyme replacement therapy; MPS II, mucopolysaccharidosis II.

^a^Based on the last recorded vital status.

^b^Four patients had an unknown diagnosis date.

^c^Age <0 years in range indicates prenatal diagnosis.

^d^MPS II was diagnosed at the age of 59.7 years in 1 patient, and MPS II was diagnosed prenatally in 1 patient; these may be considered outliers. Excluding these outliers, the age range was 0-17.8 years.

^e^Twelve patients had relatives other than siblings and/or uncles with a diagnosis of MPS II. Some patients had more than 1 relative with MPS II.

^f^The denominator is the number of patients with a positive family history.

Of the 140 patients included in this study, 108 (77.1%) had received at least 1 dose of ERT. The mean age at diagnosis was lower in patients who were subsequently treated (3.5 years) than those who remained untreated (4.8 years). Among patients in the study who had died, death occurred at a younger age in untreated patients (mean, 10.4 years; n=10) than in treated patients (15.1 years; n=8). A higher proportion of patients had also died in the untreated group than in the treated group (31.3% vs 7.4%, respectively) (Table 1). The mean (SD) time from diagnosis to start of ERT was shorter in patients in whom MPS II had been diagnosed after approval of idursulfase in 2006 than for those in whom MPS II had been diagnosed before (17.3 [17.1] and 69.2 [45.7] months, respectively). The mean (SD) follow-up was also longer for treated than for untreated patients (73.3 [49.5] and 43.3 [43.2] months, respectively). HSCT was carried out in 10 patients (7.1%); 1 of these transplants was documented as a “bone marrow graft.”

### Clinical Findings

**Figure 1.**
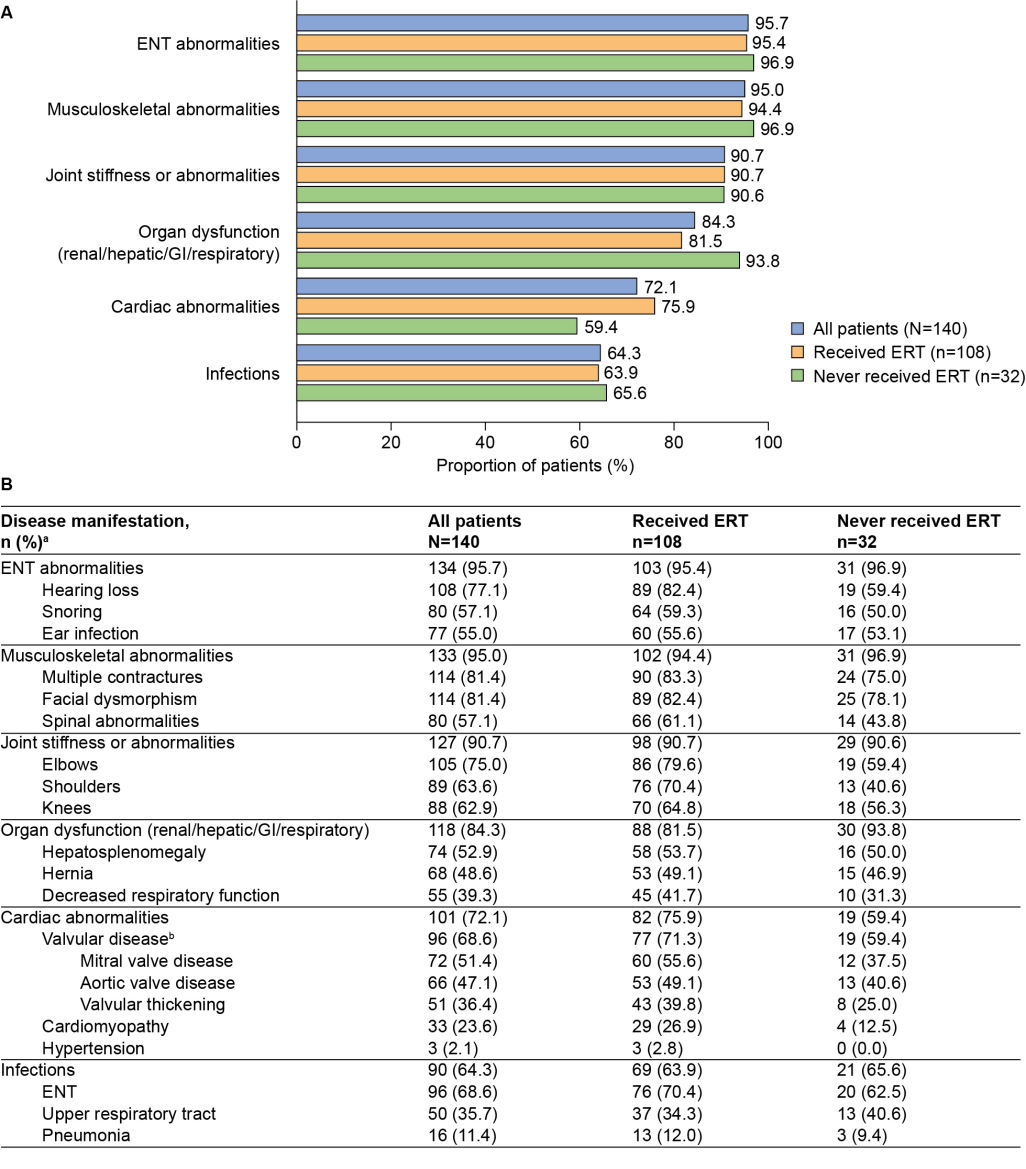
Prevalence of Common Disease Manifestations (>50% of Patients) Abbreviations: ENT, ear, nose, and throat; ERT, enzyme replacement therapy; GI, gastrointestinal. **(A)** Disease manifestations presented by category. **(B)** Disease manifestations presented by category with the 3 most common individual manifestations for each category. Results are presented for the whole analysis population (n=140) and stratified by ERT status. ^a^Data are provided for the 3 most common individual clinical manifestations for each category only. ^b^Owing to the relatively high prevalence of valvular disease in comparison to the next most common overall cardiac abnormalities, the 3 most common subtypes of valvular disease are also shown here.

The most common categories of disease manifestations (recorded in more than 50% of patients) are summarized in Figure 1. Almost all patients had ENT abnormalities (95.7%), musculoskeletal abnormalities (95.0%), and joint stiffness or abnormalities (90.7%). Organ dysfunction (84.3%), cardiac abnormalities (72.1%), and infections (64.3%) were also common. Overall, for patients with at least 1 documented infection, a mean (SD) of 0.63 (0.61) infections per patient per year was documented (**Supplementary Table S1**). Categories of disease manifestations documented in fewer than 50% of patients included decreased mobility (46.4%), skin lesions (43.6%), eye disorders (43.6%), and nerve compression (32.1%) (**Supplementary Figure S1**).

**Figure 2.**
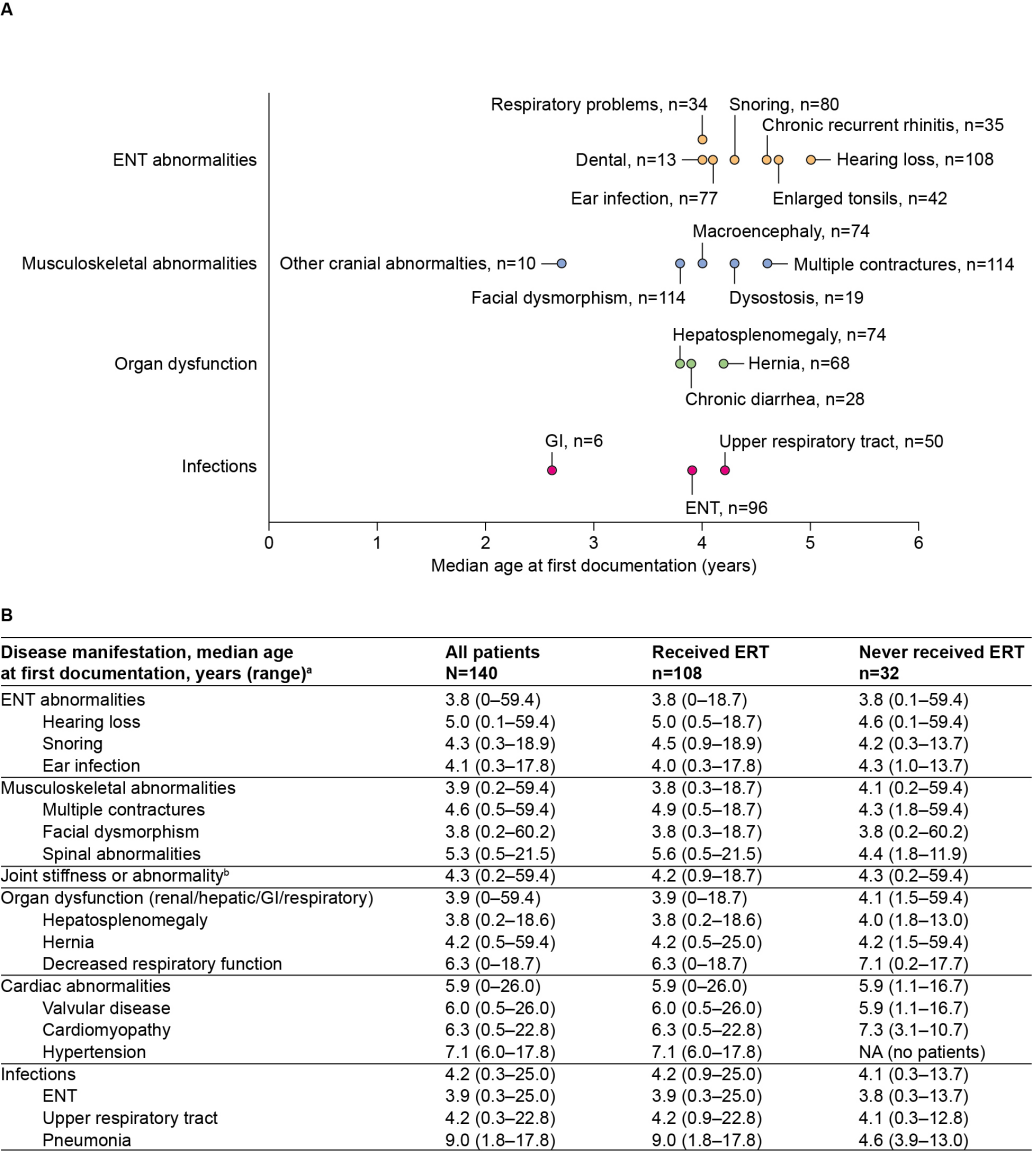
Median Age at First Documentation of Clinical Manifestations Abbreviations: ENT, ear, nose, and throat; ERT, enzyme replacement therapy; GI, gastrointestinal; NA, not applicable. **(A)** Earliest individual clinical manifestations (recorded before 5 years of age). Timeline indicates manifestations documented at a median age of up to 5 years, grouped by category; only those manifestations with data on age at first documentation available for at least 5 patients are included. **(B)** Most commonly documented clinical manifestations, for all patients and stratified by ERT status. Table indicates the age at first documentation of clinical manifestations by category and the 3 most common individual manifestations for each category. ^a^Ages are provided for the 3 most common individual clinical manifestations per category only (as identified in Figure 1B). ^b^A breakdown of age at first documentation of specific joint stiffness or abnormalities (elbows/shoulders/knees) was not available; therefore, overall category only is listed.

The majority of disease manifestations were first documented at median ages ranging from 4 to 7 years. The earliest individual disease manifestations reported for at least 5 patients are summarized in **Figure 2A.** ENT abnormalities, musculoskeletal abnormalities, and organ dysfunction (renal/hepatic/gastrointestinal/respiratory) were typically among the earliest documented disease manifestations by category, with first reports in the chart at a median age of 3.8 to 3.9 years **(Figure 2B).**

**Findings by ERT status:** Overall, the prevalence and age at first documentation of disease manifestations by category were comparable (an absolute difference of <10% in percentage prevalence and <0.5 years in median age at first documentation) between ERT-treated and untreated patients (**Figures 1 and 2**, and **Supplementary Figure S1**). However, some differences were observed in the recorded prevalence of cardiac abnormalities (75.9% of treated patients vs 59.4% of untreated patients), organ dysfunction (81.5% vs 93.8%), and nerve compression (35.2% vs 21.9%) (**Figure 1** and **Supplementary Figure S1**).

### Healthcare Resource and Supportive Service Utilization

**Figure 3.**
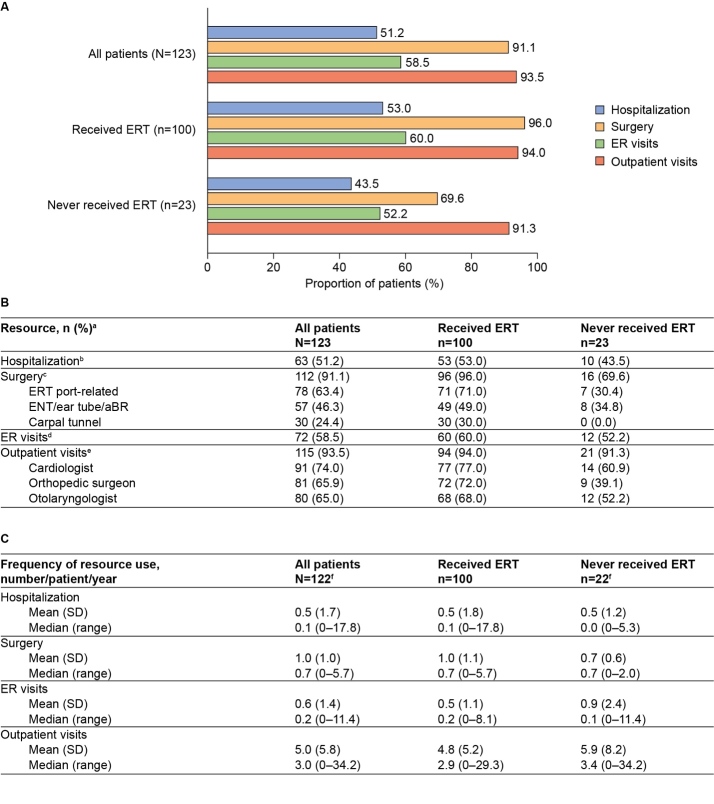
Summary of HRU (Resources Used at Least Once) Abbreviations: aBR, auditory brain stem response procedures; ED, emergency department; ENT, ear, nose, and throat; ERT, enzyme replacement therapy; HRU, healthcare resource utilization. **(A)** HRU by overall category. **(B)** HRU by overall category and the most common subtypes of resource used. **(C)** Annual resource use per patient. Results are presented for all patients and stratified by ERT status. ^a^Data are provided for the 3 most commonly used subtypes of each resource only (used at least once by the highest numbers of patients). This breakdown is not provided for hospitalization and ED visits because these data were not consistently reported. ^b^Three patients were hospitalized for ERT port-related reasons only and had no hospitalizations for other reasons. ^c^All patients who had an ERT port-related surgery also had at least 1 surgery not related to the ERT port. Further information on the reason for port surgeries in untreated patients is not available; however, reasons could include installation of a port followed by a decision not to proceed with treatment. ^d^Four patients visited the ED for ERT port-related reasons only and had no ED visits for other reasons. ^e^All patients who had an ERT port-related outpatient visit also had at least 1 visit not related to the ERT port. ^f^One patient was excluded from this analysis because data that could be used to calculate resource use frequency were unavailable.

HRU was high among the 123 patients included in this subanalysis (Figure 3). Hospitalizations and emergency department visits were reported for 51.2% and 58.5% of patients, respectively. Outpatient visits were documented for 93.5% of patients, with cardiologists being the most common specialists to have been visited at least once (74.0% of patients). Surgery was also common among the analysis population, with 91.1% of patients having undergone at least 1 surgical procedure. The most common surgeries to have been reported at least once were those related to the ERT port (63.4% of patients) and those related to the ENT system, ear tube, or auditory brain stem response (46.3% of patients) (Figure 3B).

**Figure 4.**
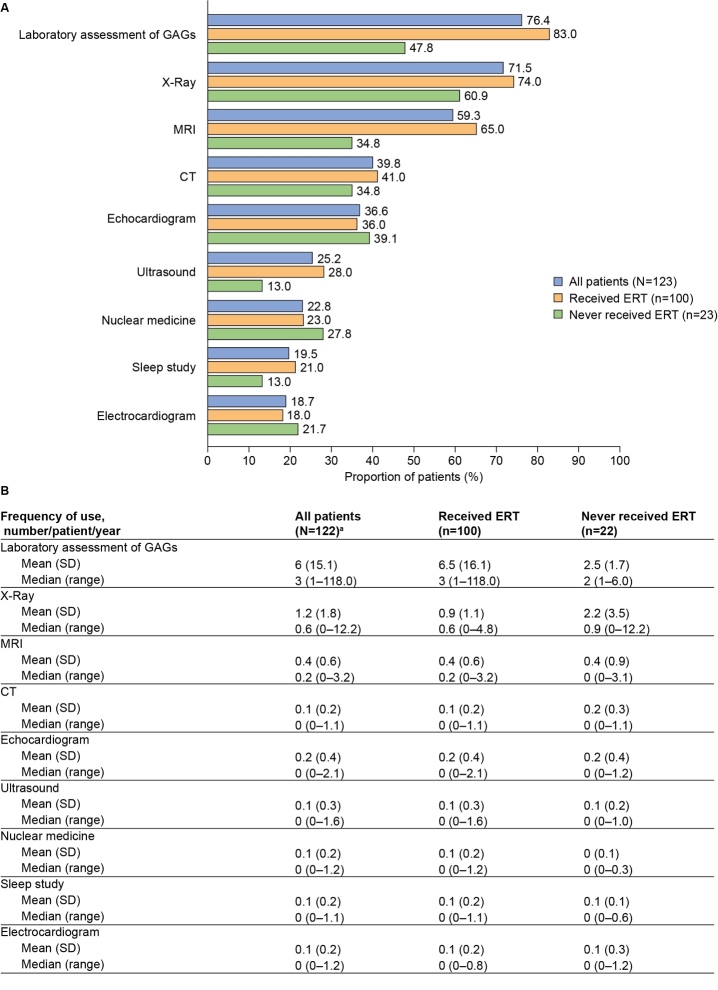
Summary of Lab Assessments of GAGs and Imaging Utilization (Imaging Procedures Performed in >15% of Patients) Abbreviations: CT, computerized tomography; ERT, enzyme replacement therapy; GAGs, glycosaminoglycans; MRI, magnetic resonance imaging. **(A)** Lab assessment of GAGs and imaging resource use by overall category. **(B)** Annual lab assessment and imaging resource use per patient. Results are presented for all patients and stratified by ERT status. ^a^One patient was excluded from this analysis because data that could be used to calculate resource use frequency were unavailable.

Laboratory testing and imaging procedures were reported for many patients, with at least 1 laboratory assessment for GAGs recorded for 76.4% of patients (n=94). Imaging was also common in this analysis population (87.8%), most commonly X-ray (71.5%) and magnetic resonance imaging (59.3%) (Figure 4A).

**Figure 5.**
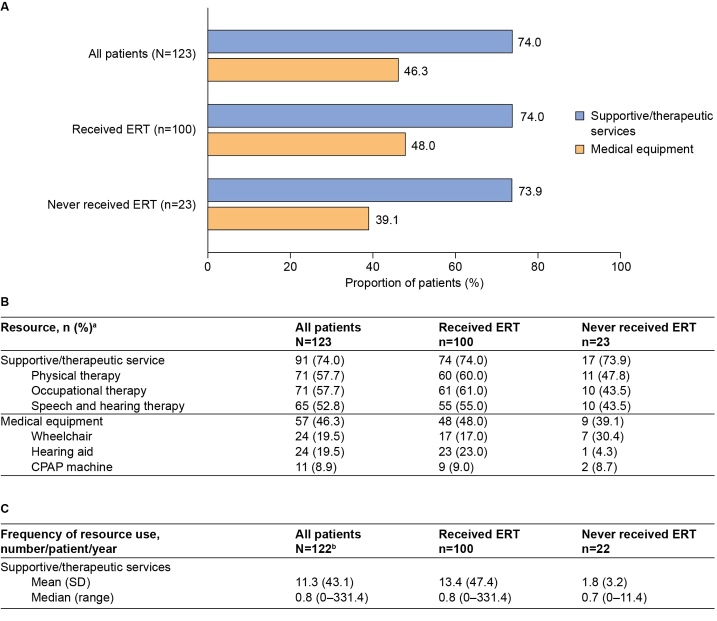
Summary of Supportive and Therapeutic Service Utilization (Resources Used at Least Once) Abbreviations: CPAP, continuous positive airway pressure; ERT, enzyme replacement therapy. **(A)** Supportive/therapeutic services and medical equipment utilization by overall category. **(B)** Supportive/therapeutic services and medical equipment utilization by overall category and the most common subtypes of resource used. **(C)** Annual resource use per patient. Results are presented for all patients and stratified by ERT status. ^a^Data are provided for the 3 most commonly used subtypes of each resource only (used at least once by the highest numbers of each patients).

Use of supportive and therapeutic services was extensive, with at least 1 use reported for 74.0% of patients (Figure 5A). The most commonly used services included physical therapy (57.7%), occupational therapy (57.7%), and speech and hearing therapy (52.8%). Moreover, use of medical equipment was recorded for 46.3% of patients; the most commonly used pieces of medical equipment were wheelchairs (19.5%) and hearing aids (19.5%) (Figure 5B).

The annual frequency of healthcare resource and supportive service use was also evaluated (n=122). Patients had a median (range) of 0.1 (0-17.8) hospitalizations, 0.2 (0-11.4) emergency department visits, and 3.0 (0-34.2) outpatient visits per patient per year (Figure 3C). Patients spent a median of 0.1 (0-247.9) days per year in hospital. For supportive services (Figure 5C), the range of annual frequency of use was very large (median, 0.8 [range, 0-331.4] uses per patient per year).

**Findings by ERT status:** With some notable exceptions, the prevalence and frequency of use of healthcare resources and supportive services were generally similar in treated and untreated patients. A key exception was surgeries, which were more commonly reported for patients who had received ERT (96.0%) than for those who had not (69.6%). The potential influence of ERT port-related surgeries on this difference was explored; however, in all patients for whom 1 or more surgeries were reported, at least 1 of those surgeries was not port-related. Laboratory assessments of GAGs were more common in treated patients (83.0%) than in untreated patients (47.8%); the annual median (range) frequency of measurements was also higher in treated than in untreated patients (3.0 [1-118.0] vs 2.0 [1-6.0], respectively) (Figure 4B).

### Sensitivity Analysis: Influence of HSCT

Overall, clinical findings and rates of HRU were similar when the 10 patients who underwent HSCT were excluded from the analysis (data not shown).

## DISCUSSION

We analyzed a large amount of data from patients with MPS II across 19 sites in the United States. The findings add to the body of available real-world evidence on the nature of this rare disease and demonstrate that patients with MPS II experience a complex and substantial clinical burden, driving a high level of associated HRU. Furthermore, the observed clinical manifestations and high rate of resource use in both treated and untreated patients highlight the need for regular monitoring and multidisciplinary care for all patients with MPS II, irrespective of treatment status.

The clinical findings outlined in this study are broadly in line with previous reports of the natural history of MPS II, suggesting that the patients included in this analysis are representative of MPS II patients.^8,9,12,22^ Organ dysfunction and disease manifestations associated with the ENT and musculoskeletal systems were commonly recorded among patients in this study. This is in line with previous reports,^8,9,12^ including an analysis of registry data^12^ in which a 95% prevalence was recorded for facial dysmorphism, similar to our observed prevalence of 81%. The prevalence recorded for hepatosplenomegaly was 89%,^12^ which was higher than the 53% prevalence observed in this study; organ dysfunction overall, however, was recorded with a similar prevalence of 84%. Furthermore, in agreement with previous estimates, approximately two-thirds of patients in this study had cognitive impairment recorded.^6,8,9,23^

ENT abnormalities, organ dysfunction, and musculoskeletal abnormalities were recorded early in most patient charts, which is generally consistent with previous reports on the natural history of MPS II.^6,12,22^ However, the majority of disease manifestations in this study were documented at a median age of 4‑7 years, which is later than previously reported. For example, in a study of registry data, the median age at onset of hepatosplenomegaly was 2.8 years compared with 3.8 years in this study.^12^ It is important to note that this analysis reports data from patient charts, thereby capturing the age at first documentation of disease manifestations by a specialist clinic rather than age at onset. Furthermore, these disparities in age at onset may also reflect further differences in the study design and inclusion criteria and, as a result, the overall study population.

Overall, the prevalence of particular disease manifestations by category and age at first documentation were comparable between treated and untreated patients. Some slight differences (>10% absolute difference in percentage prevalence) were observed for cardiac abnormalities and nerve compression, both of which were recorded in a greater proportion of treated than untreated patients. On the other hand, organ dysfunction appeared to be more prevalent in untreated patients than in treated patients. One reason for these differences may be that severely affected patients are more likely to receive treatment. Furthermore, temporality was not taken into consideration in this study, so clinical findings may have been recorded before or after treatment initiation.

It is clear from the clinical findings reported above that, irrespective of treatment status, patients with MPS II experience significant disease burden, and as a result HRU is also extensive. As with other similar studies, a limitation of this analysis was that data were from a single country. However, resource use among this study population was similar to that previously reported in France and in a separate US study, despite differences in methodology.^15,17^ For example, in line with the results of the US study, cardiologists were the most commonly visited specialist. Furthermore, nearly half of patients had 1 or more day surgeries and more than half of patients had been hospitalized within the preceding 12 months, corroborating our findings of significant HRU amongst this US patient population.^17^

Patients underwent frequent surgeries: nearly all patients (91%) in this study underwent at least one surgery. This aligns with a previous report by Mendelsohn et al,^24^ which reported surgical interventions in 84% of that study population. Given that more than three-quarters of patients in this study had received ERT, it is perhaps unsurprising that port-related procedures were the most common. Ear tube procedures, tonsillectomy or adenoidectomy, and carpal tunnel corrections were also frequently recorded. Hernia repair was among the more commonly recorded surgeries in previous studies; however, it was reported in less than 20% of patients in this study (compared with 50% and 57% as reported by Mendelsohn et al^24^ and Lin et al,^25^ respectively). This difference may relate to the older mean age at first documentation of hernia in this population or differences in the study populations.

HRU was generally comparable among treated and untreated patients, although surgery was more commonly reported for treated patients. ERT port-related surgeries were common; however, these can be excluded as the reason for this difference, because all patients who had undergone at least 1 surgical procedure had also undergone at least 1 surgery that was not related to the ERT port.

The highlighted differences in both the prevalence of surgery and some aspects of resource use may reflect that treated patients are likely to live longer and to be more closely monitored, so they may access healthcare services more frequently. Conversely, patients who are less severely affected by the disease may experience a delay in receiving an MPS II diagnosis and treatment, and may be less likely to visit the hospital than those with more severe disease.

### Limitations

As with most studies of MPS II,^26^ comparisons between treated and untreated patient groups were limited by the low number of untreated patients and the heterogeneity of the patient population. The varying duration of ERT and changes in the overall standards of clinical care since the introduction of idursulfase were additional confounding factors. The study design had limitations, such as use of physician notes without access to tests to validate comorbidities, variability in the available recorded data, and missing information on the actual timing of some recorded events (including in relation to ERT initiation). However, it also had several strengths, such as the relatively large patient numbers for a rare disease, long duration of follow-up, and the extended time period of the study. In addition, the results of real-world data analyses such as this will provide informative indicators of HRU for use in economic modeling analyses, which are currently lacking in the literature for MPS II. These analyses can also take into account important factors among this patient population, such as the impact of disease severity on treatment benefit. Overall, our analysis provides a long-term view of disease burden and HRU among patients with MPS II in the United States.

## CONCLUSIONS

MPS II is a chronic and debilitating condition. Our findings contribute to the growing understanding of the natural history of this disease and demonstrate that patients experience significant disease burden. As may be expected owing to the multisystemic nature of the disease and the need for regular follow-up and evaluation, we show that the healthcare needs of patients with MPS II are extensive. Irrespective of treatment status, our findings demonstrate that surgical procedures, outpatient visits, and use of supportive services are common among the patient population. Increased understanding of the common manifestations of this disease and the associated HRU may help to inform more effective disease management and, ultimately, contribute to improvements in patient care.

### Author Contributions

OA contributed to study conception, design and data interpretation. KM and SS performed the chart review analysis and contributed to study design and data interpretation. OA, DA, and KSY contributed to study conception, design and data interpretation.

### Disclosures

OA is a full-time employee of Takeda Development Center Americas, Inc. and stockholder of Takeda Pharmaceutical Company Limited. KM was an employee of ICON plc, a contract research organization contracted for this chart review, at the time this study was conducted. SS was an employee of ICON plc, a contract research organization contracted for this chart review, at the time this study was conducted. DA was a full-time employee of Takeda Development Centers Americas, Inc. at the time this manuscript was prepared and a stockholder of Takeda Pharmaceutical Company Limited. KSY was a full-time employee of Takeda Development Centers Americas, Inc. and a stockholder of Takeda Pharmaceutical Company Limited.

### Availability of Data and Materials

The datasets, including redacted study protocol, redacted statistical analysis plan, and individual participants’ data supporting the results reported in this article, will be made available within 3 months from initial request to researchers who provide a methodologically sound proposal. The data will be provided after its de-identification in compliance with applicable privacy laws, data protection, and requirements for consent and anonymization.

## Supplementary Material

Online Supplemental Material

Online Supplemental Material
