# Supplementary material for: Clinical Characteristics and Healthcare Resource Utilization for Patients with Mucopolysaccharidosis II (MPS II) in the United States: A Retrospective Chart Review
Source: J Health Econ Outcomes Res. 2022 May 12;9(1):117–27. doi: 10.36469/jheor.2022.33801 (PMC9098230; doi:10.36469/jheor.2022.33801)
Supplement: Online Supplemental Material [file jheor_2022_9_1_33801_89376.pdf]

Journal of Health Economics and Outcomes Research

Online Supplementary Material

Ayodele O, Müller K, Setayeshgar S, Alexanderian D, Yee KS. Clinical Characteristics and Healthcare Resource Utilization for Patients with Mucopolysaccharidosis II (MPS II) in the United States: A Retrospective Chart Review. *JHEOR*. **2022;9(1):117-127.** doi:10.36469/jheor.2022.33801

Figure S1: Prevalence of Common Disease Manifestations (<50% of Patients) in the Analysis Population, Stratified by Enzyme Replacement Treatment Status

Table S1: Mean (SD) Number of Infections Documented per Year

This supplementary material has been provided by the authors to give readers additional information about their work.

This is an open-access article distributed under the terms of the Creative Commons Attribution 4.0 International License (CCBY-4.0). View this license's legal deed at http://creativecommons.org/licenses/by/4.0 and legal code at http://creativecommons.org/licenses/by/4.0/legalcode for more information.

Figure S1. Prevalence of Common Disease Manifestations (<50% of Patients) in the Analysis Population (n=140) and Stratified by ERT Status

A

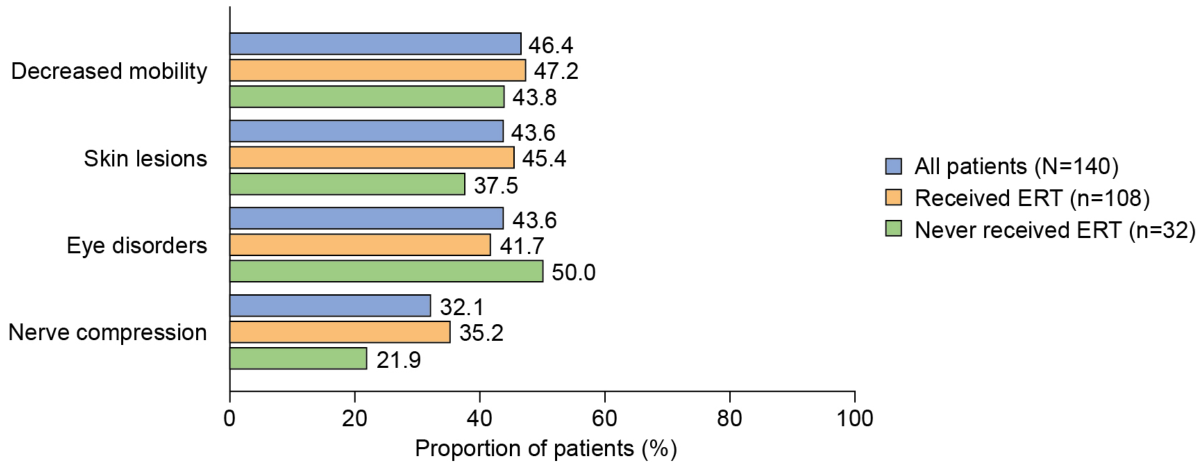

B

Disease manifestation, n (%)[a]	All patients N=140	Received ERT n=108	Never received ERT n=32
Decreased mobility	65 (46.4)	51 (47.2)	14 (43.8)
Difficulties with ambulation	57 (40.7)	45 (41.7)	12 (37.5)
Difficulties with standing up from seated	41 (29.3)	32 (29.6)	9 (28.1)
Gait toe walk	15 (10.7)	13 (12.0)	2 (6.3)
Skin lesions	61 (43.6)	49 (45.4)	12 (37.5)
Mongolian spots	23 (16.4)	20 (18.5)	3 (9.4)
Pebbling	16 (11.4)	12 (11.1)	4 (12.5)
Ichthyosis	14 (10.0)	12 (11.1)	2 (6.3)
Eczema	14 (10.0)	11 (10.2)	3 (9.4)
Eye disorders	61 (43.6)	45 (41.7)	16 (50.0)
Refractive errors	45 (32.1)	36 (33.3)	9 (28.1)
Other eye disorders[b]	20 (14.3)	12 (11.1)	8 (25.0)
Strabismus	11 (7.9)	7 (6.5)	4 (12.5)
Nerve compression	45 (32.1)	38 (35.2)	7 (21.9)
Carpal tunnel syndrome	37 (26.4)	34 (31.5)	3 (9.4)
Spinal cord compression	13 (9.3)	11 (10.2)	2 (6.3)
Other nerve compression	9 (6.4)	5 (4.6)	4 (12.5)

(A) Disease manifestations by category. **(B)** Disease manifestations by category and the 3 most common individual manifestations for each category.

Abbreviation: ERT, enzyme replacement therapy.

[a]Data are provided for the three most common individual clinical manifestations for each category only.

[b]All other recorded eye disorders excluding retinal regeneration, corneal opacity, ocular hypertrophy, glaucoma, and blindness.

Table S1. Mean (SD) Number of Infections Documented per Year

	All Patients (n=140)	Received ERT (n=108)	Never Received ERT (n=32)
Number of infections documented/year	0.63 (0.61)	0.56 (0.5)	0.89 (0.8)
Upper respiratory tract	0.44 (0.41)	0.36 (0.32)	0.7 (0.52)
ENT	0.36 (0.28)	0.32 (0.25)	0.51 (0.34)
Pneumonia	0.25 (0.24)	0.19 (0.12)	0.49 (0.47)
Port line	0.18 (0.11)	0.19 (0.11)	0.11 (0.0)
GI	0.16 (0.1)	0.16 (0.1)	0 (0.0)
Fungal or yeast	0.42 (0.54)	0.47 (0.58)	0.17 (0.0)
Other	0.17 (0.07)	0.16 (0.07)	0.17 (0.12)

Abbreviations: ENT, ear, nose, and throat; ERT, enzyme replacement therapy; GI, gastrointestinal.
